# Preventive Effect of *Lactobacillus fermentum* CQPC08 on 4-Nitroquineline-1-Oxide Induced Tongue Cancer in C57BL/6 Mice

**DOI:** 10.3390/foods8030093

**Published:** 2019-03-11

**Authors:** Bihui Liu, Jing Zhang, Ruokun Yi, Xianrong Zhou, Xingyao Long, Yanni Pan, Xin Zhao

**Affiliations:** 1 Chongqing Collaborative Innovation Center for Functional Food, Chongqing University of Education, Chongqing 400067, China; liubh@foods.ac.cn (B.L.); yirk@cque.edu.cn (R.Y.); zhouxr@foods.ac.cn (X.Z.); longyaoyao@foods.ac.cn (X.L.); panyanni@foods.ac.cn (Y.P.); 2 Chongqing Engineering Research Center of Functional Food, Chongqing University of Education, Chongqing 400067, China; 3 Chongqing Engineering Laboratory for Research and Development of Functional Food, Chongqing University of Education, Chongqing 400067, China; 4 College of Biological and Chemical Engineering, Chongqing University of Education, Chongqing 400067, China; 5 Environment and Quality Inspection College, Chongqing Chemical Industry Vocational College, Chongqing 401228, China; zjinger0810@126.com; 6 College of Food Science, Southwest University, Chongqing 400715, China; 7 Department of Food Science and Biotechnology, Cha University, Gyeongghi-do, Seongnam 13488, Korea

**Keywords:** *Lactobacillus fermentum* CQPC08, 4-nitroquineline-1-oxide, tongue cancer, C57BL/6 mice, protein

## Abstract

*Lactobacillus fermentum* CQPC08 (LF-CQPC08) is a newly discovered strain of bacteria isolated and identified from traditional pickled vegetables in Sichuan, China. We used 4-nitroquinoline 1-oxide to establish an experimental tongue cancer mouse model to evaluate the preventive effect of LF-CQPC08 on tongue cancer in vivo. *Lactobacillus delbruechii* subsp. *bulgaricus*, is a common commercial strain and is used as a positive control to compare the effect with LF-CQPC08. The preventive strength and mechanism of LF-CQPC08 on tongue cancer were determined by measuring the biochemical indicators in mouse serum and tissues. Our results showed LF-CQPC08 inhibits the decline of splenic index, thymus index, percentage of phagocytic macrophages, and phagocytic index effectively. LF-CQPC08 also increased levels of mouse serum granulocyte-colony stimulating factor (G-CSF), granulocyte-macrophage-CSF (GM-CSF), immunoglobulin (Ig)G, IgM levels of serum interleukin (IL)-4, IL-12, tumor necrosis factor-alpha, and interferon-gamma levels, thereby inhibiting the decline in immunity caused by tongue cancer. It also increased the activity levels of superoxide dismutase and glutathione peroxidase and decreased the levels of malondialdehyde in the tissues of the tongue cancer mouse model, thereby suppressing the oxidative stress damage in the tissue caused by tongue cancer. Through quantitative PCR, LF-CQPC08 upregulated the mRNA expression of nuclear factor-erythroid 2 related factor 2 (Nrf2), heme oxygenase-1 (HO-1), glutathione-S-transferases-π (GST-π), and Bcl-2-associated X protein (Bax), and downregulated the mRNA expression of p53, p63, p73, phosphatase and tensin homolog (PTEN), B-cell lymphoma 2 (Bcl-2) and B-cell lymphoma-extra large (Bcl-xL) in the tongue tissues of the tongue cancer mouse. These results indicated that LF-CQPC08 reduced the influence of tongue cancer on the immune system and oxidative balance and improved the immunity and enhanced antioxidant capacity of the mouse model, thereby preventing tongue cancer. LF-CQPC08 could be used as a microbial resource with a preventive effect on tongue cancer.

## 1. Introduction

Fermentation has been used to prolong the storage life of vegetables in many cultures, including China. Sichuan-style pickled vegetables are as famous as French cornichons and German sauerkraut [1]. Sichuan-style pickling relies mainly on the fermentation of lactic acid bacteria to produce high concentrations of lactic acid instead of on the osmotic pressure of salt to inhibit putrefying microorganisms. Low-concentration saline is used to preserve fresh vegetables for Sichuan-style pickling. This is followed by fermentation by lactic acid bacteria. So long as the product is in an airtight environment and the lactic acid reaches a certain concentration, long-term preservation and flavorful taste can be achieved [2]. Because Sichuan-style pickling involves reusing the salt solution, long-term and repeated fermentation produces a complex microbial system containing abundant bacterial flora, including such known species as *Lactobacillus brevis*, *L. plantarum*, ethanol-resistant *Fasciococcus*, *L. casei*, *L. pentosus*, *L. sakei, L. alimentarius*, and *Leuconostoc mesenteroides* [3,4,5]. Because the fermentation conditions of the Sichuan-style pickled vegetables are different from those of other fermented foods, the microorganisms in the Sichuan-style pickled vegetables include special strains with their own characteristics including resistance to acid [6].

The great numbers of lactic acid bacteria do not only play a key role in the taste and quality of pickled vegetables but also contribute to various biological activities [7]. Lactic acid bacteria maintain the balance of microbial ecology in human body, improve the digestibility and utilization of food in the gastrointestinal tract, inhibit the growth and reproduction of spoilage bacteria in the intestine, and produce nutrients for the body to use. They also reduce serum cholesterol and the effects of toxin stimulation on body tissue development. Lactic acid bacteria also play a probiotic role in regulating the nutritional status of the body, improving the physiological function of the body, avoiding cell infection, improving the efficacy of drugs, alleviating the effects of toxic substances on the body, promoting immune response, preventing tumorigenesis and slowing down aging [8,9,10,11,12]. Reduction of probiotics in the body can lead to abnormalities in the body. Therefore, maintaining the normal level of probiotics in the body plays an important role in human health. Lactic acid bacteria have a good effect on maintenance of normal microbial ecology in the body, so they have been extensively used as probiotics in food, medicine, and the pharmaceutical industry [13].

The immune system is an important part of how the human body defends itself from foreign pathogens, and it can distinguish invading harmful substances such as bacteria, virus, molds and other pathogenic microorganisms from its own cells and eliminate them [14]. During the early stage of cancer, which weakens the immune system, cancer cells are only rarely detected and eliminated, which gives them the opportunity to develop into tumors. The immunity of cancer patients also affects the speed of cancer progression and treatment outcomes. Therefore, improving the immunity of cancer patients may increase the success rate of anticancer therapies [15]. The immunomodulatory effects of lactic acid bacteria on the human body have two main aspects: (1) regulation of non-specific immunity; and (2) regulation of specific immunity. Lactic acid bacteria regulate specific and non-specific immune responses in the body to facilitate maintenance of the normal level of immune functions in the body and play a very important role in nutrition, biological barriers, anti-tumor functions, and other probiotic functions of the body [16].

Oxidative stress is closely related to the occurrence of disease conditions, such as tumors, inflammation, neurodegeneration, and aging. Under normal circumstances, oxidative metabolism in the bodies of living beings produces a small quantity of free radicals, which can be eliminated by the antioxidant system of the body to maintain redox balance. However, under the influence of some injury factors, accumulation of large quantities of free radicals are induced, thereby forming an imbalance of oxidation and antioxidation, which is known as oxidative stress and is directly related to the onset of cancers [17].

Tongue cancer is a malignant tumor originating in the anterior part of the tongue and is one of the most common malignant tumors in the oral and maxillofacial region, accounting for 0.8–2.0% of systemic cancer, 5–15.5% of head and neck cancer, and 32.3% of oral cancer; it is ranked first in oral cancer [18]. A large number of microorganisms are found in the oral cavity. Studies on the inhibitory effects of lactic acid bacteria on tongue cancer through immunomodulation in the oral cavity and in the body are rarely reported. Lactic acid bacteria have not been isolated from Sichuan pickled vegetables by searching references. By comparison with Gene Bank, LF-CQPC08 (sequence: TTAGGCGGTGGCTCCTAAAGGTTACCCCACCGACTTTGGGTGTTAAAACTCTCATGGTGTGACGGGCGGTGTGTACAAGGCCCGGGAACGTATTCACCGCGGCATGCTGATCCGCGATTACTAGCGATTCCGACTTCGTGCAGGCGAGTTGCAGCCTGCAGTCCGAACTGAGAACGGTTTTAAGAGATTTGCTTGCCCTCGCGAGTTCGCGACTCGTTGTACCGTCCATTGTAGCACGTGTGTAGCCCAGGTCATAAGGGGCATGATGATCTGACGTCGTCCCCACCTTCCTCCGGTTTGTCACCGGCAGTCTCACTAGAGTGCCCAACTTAATGCTGGCAACTAGTAACAAGGGTTGCGCTCGTTGCGGGACTTAACCCAACATCTCACGACACGAGCTGACGACGACCATGCACCACCTGTCATTGCGTTCCCGAAGGAAACGCCCTATCTCTAGGGTTGGCGCAAGATGTCAAGACCTGGTAAGGTTCTTCGCGTAGCTTCGAATTAAACCACATGCTCCACCGCTTGTGCGGGCCCCCGTCAATTCCTTTGAGTTTCAACCTTGCGGTCGTACTCCCCAGGCGGAGTGCTTAATGCGTTAGCTCCGGCACTGAAGGGCGGAAACCCTCCAACACCTAGCACTCATCGTTTACGGCATGGACTACCAGGGTATCTAATCCTGTTCGCTACCCATGCTTTCGAGTCTCAGCGTCAGTTGCAGACCAGGTAGCCGCCTTCGCCACTGGTGTTCTTCCATATATCTACGCATTCCACCGCTACACATGGAGTTCCACTACCCTCTTCTGCACTCAAGTTATCCAGTTTCCGATGCACTTCTCCGGTTAAGCCGAAGGCTTTCACATCAGACTTAGAAAACCGCCTGCACTCTCTTTACGCCCAATAAATCCGGATAACGCTTGCCACCTACGTATTACCGCGGCTGCTGGCACGTAGTTAGCCGTGACTTTCTGGTTAAATACCGTCAACGTATGAACAGTTACTCTCATACGTGTTCTTCTTTAACAACAGAGCTTTACGAGCCGAAACCCTTCTTCACTCACGCGGTGTTGCTCCATCAGGCTTGCGCCCATTGTGGAAGATTCCCTACTGCTGCCTCCCGTAGGAGTATGGGCCGTGTCTCAGTCCCATTGTGGCCGATCAGTCTCTCAACTCGGCTATGCATCATCGCCTTGGTAGGCCGTTACCCCACCAACAAGCTAATGCACCGCAGGTCCATCCAGAAGTGATAGCGAGAAGCCATCTTTTAAGCGTTGTTCATGCGAACAACGTTGTTATGCGGTATTAGCATCTGTTTCCAAATGTTGTCCCCCGCTTCTGGGCAGGTTACCTACGTGTTACTCACCCGTCCGCCACTCGTTGGCGACCAAAATCAATCAGGTGCAAGCACCATCAATCAA) was initially considered as a newly discovered lactic acid bacteria. After treatment with LF-CQPC08 for one month by lavage, the IgG serum level was 1.67 times higher than that in mice without LF-CQPC08; the stain showed the immunomodulatory effect. This study evaluated the inhibitory effects of the newly discovered, lactic acid bacteria LF-CQPC08 on tongue cancer mouse model and explored the mechanism underlying the action of LF-CQPC08 on 4-nitroquinoline 1-oxide (4NQO)-induced tongue cancer through immunomodulation. The experimental results of this study may facilitate definition of the functional role of LF-CQPC08 and indicate new areas of tongue cancer inhibition through probiotics.

## 2. Materials and Methods

### 2.1. Isolation and Identification of the Lactic Acid Bacteria from Pickled Vegetables

One milliliter of pickle juice collected from the commercially available pickled vegetables sold in Chongqing City, Sichuan Province, China was used for gradient dilution with sterile saline solution to 10^−6^ times of the stock solution, followed by pipetting and inoculation of 100 μL of 10^−4^, 10^−5^, 10^−6^ of the stock solution separately onto agar plate to further incubate at 37 °C for 48 h in order to subsequently observe and record the morphologies of colony formation. The colonies on the agar plate, in different forms, were picked and streaked on new agar plate individually to continue incubation at 37 °C for 48 h, and these procedures were repeated two to three times until pure single colonies of consistent morphology were obtained. The pure colonies on the agar plates were picked, inoculated in 5 mL De Man, Rogosa and Sharpe (MRS) broth, and incubated at 37 °C for 24 h. Subsequently, 1 mL of the above bacteria-containing culture solution was pipetted into a sterile centrifuge tube for 4000 rpm centrifugation for 10 min. After discarding the supernatant, the bacterial pellet was resuspended in sterile physiological saline for gram staining. The purified target bacterial strain after microscopic examination was cultured in MRS broth 37 °C for 24 h. We then isolated the bacterial DNA using a genomic DNA extraction kit. PCR was performed to amplify 16S rDNA, and the PCR products were identified by agarose gel electrophoresis. The PCR system contained 1 μL upstream primer 7F (5′-AGAGTTTGATCCTGGCTCAG-3′), 1 μL downstream primer 495R (5′-CTACGGCTACCTTGTTACGA-3′), 21.25 μL×Taq plus buffer, 1 μL DNA template, and double-distilled water to top up the final volume to 25 μL. Sterile ultrapure water was used to replace DNA template to prepare the negative control PCR group. The amplification conditions were: 94 °C denaturation for 5 min; 29 cycles of 94 °C denaturation for 30 s, 55 °C annealing for 30 s, 72 °C elongation for 1 min; and 72 °C elongation for 5 min. Here, 5 μL PCR product was subjected to 1.5% agarose gel electrophoresis under the condition of 110 V for 45 min. Finally, the 16S rRNA PCR products were sequenced [19]. Sequences of the bacterial strains were aligned using the BLAST in NCBI website.

### 2.2. Animal Experiments

Fifty 10-week female specific-pathogen-free (SPF) grade C57BL/6 mice were purchased from Chongqing Medical University (Chongqing City, China) and housed in an SPF animal facility with a room temperature of 18–25 °C, humidity of 30–50%, standard feed, and a 24 h light-dark cycle. After a week of adaptation, the mice were evenly divided into five groups: the Normal group, Control group, LDSB gavage group, low-dose LF-CQPC08 gavage (LF-CQPC08-L) group, and high-dose LF-CQPC08 gavage (LF-CQPC08-H) group, with 10 mice per group. Mice in the Control group LDSB group, LF-CQPC08-L group and LF-CQPC08-H were given water containing 100 μg/mL 4NQO for 16 consecutive weeks. In addition, mice in the LDSB group were intragastrically administered 1.0 × 10^9^ CFU/kg LDSB for 16 consecutive weeks, and LF-CQPC08-L, LF-CQPC08-H groups were intragastrically administered 1.0 × 10^8^ LF-CQPC08, 1.0 × 10^9^ CFU/kg LF-CQPC08, respectively, once a day for 16 consecutive weeks. Then all animals were fasted for 24 h and sacrificed by cervical dislocation [20] after the corresponding treatments to withdraw the blood from heart and dissect the tongue for subsequent experiments. The spleen and thymus of each mouse were dissected and weighed separately to calculate the viscera (spleen and thymus) index (viscera (spleen and thymus) index = viscera (spleen and thymus) weight (g)/mouse body weight (g) × 100), respectively. This study was conducted in accordance with the Declaration of Helsinki, and the protocol was approved by the Ethics Committee of Chongqing Collaborative Innovation Center for Functional Food (201807001B).

### 2.3. Mouse Non-Specific Immune Function Assay

The peritoneal fluid was aspirated from each of the sacrificed mice to prepare a peritoneal macrophage smear sample, which was stained with Giemsa stain for the observation of ability of macrophages phagocytosing chicken erythrocytes under a microscope. The phagocytosis percentage and phagocytic index were calculated according to the following formulas: percentage of phagocytic macrophages (%) = the number of phagocytic macrophages/total number of counted macrophages × 100%; and phagocytic index = Total number of phagocytosed chicken erythrocytes/total number of counted macrophages × 100% [21].

### 2.4. Determination Serum IL-4, IL-12, TNF-A, IFN-Γ, G-CSF, GM-CSF, IgG and IgM Levels in Mice

The obtained blood was centrifuged at 4000 rpm for 10 min and then the upper serum was taken. The levels of IL-4, IL-12, IFN-γ, TNF-a (Abcam, Cambridge, MA, USA) [22] and G-CSF, GM-CSF, IgG, IgM (Thermo Fisher Scientific, Inc., Waltham, MA, USA) [23] in the mice were determined according to the kit instructions.

### 2.5. Determination of Oxidative Index of Mouse Tongue Tissue

From each mouse, 0.1 g of tongue tissue was homogenized with 9 times of saline, and then centrifuged at 4000 rpm for 10 min. The supernatant was taken and the SOD, GSH-Px activities and the MDA level of tongue tissue were determined according to the kit instructions (Nanjing Jiancheng Bioengineering Institute, Nanjing, China).

### 2.6. Quantitative PCR (qPCR) Assay

The fresh mouse tongue tissues were homogenized, followed by using RNAzol reagent to extract the total RNAs in the tongue tissues and dilute the total RNAs to 1 μg/μL according to the instructions of the transcription kit (RNAsimple total RNA extraction kit, Tiangen Biotech (Beijing) Co., Ltd., Beijing, China). The concentration of total RNA was detected at 260 and 280 nm using a micro-ultraviolet spectrophotometer (Nano 300, Aosheng, Hanzhou, Zhejiang, China). For generation of cDNA, the 9 μL oligo primer DT and 1 μL (1 μg/μL) RNA solution were mixed and the mixture solution was putt into the PCR instrument (ProFlex PCR, Thermo Fisher Scientific, Inc.) for a thermal cycle of 65 °C, 5 min, then 4 μL 5×Reaction Buffer, 1 μL Ribolock RNase inhibitor, 2 μL 100 mM NTP mix and 1 μL RevertAid RT were added to the above solution. After mixing, the cDNA was produced by PCR instrument at 42 °C for 60 min and 70 °C for 5 min. PCR reactions were done in a total volume of 20 μL reaction solution, the reaction solution included 2 μL cDNA, 10 μL SYBR Green PCR Master Mix (Thermo Fisher Scientific), 1 μL forward primer solution, 1 μL reverse primer solution (4 μM) (Table 1, Thermo Fisher Scientific, Inc.) and 6 μL distilled water [23]. The reaction solution was reacted according to the following conditions: 95 °C denaturation for 60 s; 40 cycles of 95 °C denaturation for 15 s, 55 °C annealing for 30 s, 72 °C elongation for 35 s; and eventually tested at 95 °C for 30 s and 55 °C for 35 s. GAPDH was used as an internal reference of the qPCR, and the 2^−ΔΔCt^ method was used to calculate the relative gene expression in this study [24].

### 2.7. Statistical Analysis

We conducted three parallel experiments on the serum and tissue assays of each mouse to obtain the average values. SAS9.1 statistical software was used for data analysis. First the homogeneity of variances was tested using the Bartlett test, and the univariate normality test was tested by skewness-kurtosis test. The post-hoc test was used to analyze whether there were significant differences between any two groups data in the experiment by one-way ANOVA analysis [25].

## 3. Results

### 3.1. LF-CQPC08 Isolation and Identification

The microorganism was isolated from natural fermented Sichuan pickles (unsterilized; 30 °C fermentation for 7 days; made from Chinese cabbage, salt and water). The morphologies of strain and colony of bacteria were observed. They were mainly milky white in color and round in shape, with neat and smooth edges. The initial microscopic results of gram staining showed that the positive strain was gram-positive. Under 100× magnification, the morphologies of the gram-positive bacterial strain included long and short rods, without budding. The results of agarose gel electrophoresis of the 16S ribosomal DNA (16S rRNA) showed no band in the negative control group, confirming no contamination in the PCR. A band approximately 1500 bp long was observed in the lane of the bacterial strain, which was consistent with the anticipated size of the amplified fragment. The basic local alignment search tool (BLAST) in NCBI was used to analyze the aligned sequences, and the results showed that the strain numbered LF-CQPC08 was *Lactobacillus fermentum* in lactic acid bacteria, and shared 99% homology with the known lactic acid bacteria in the Gene Bank database (Gene Bank accession number: NC_010610.1). The strain was named *L. fermentum* CQPC08 (LF-CQPC08), with the patented species preservation number as CGMCC No. 14957 (China General Microbiological Culture Collection Center, Beijing, China).

### 3.2. Effect of Lactobacillus Fermentum CQPC08 on Immune Organ Index in Mice

**Figure 1:**
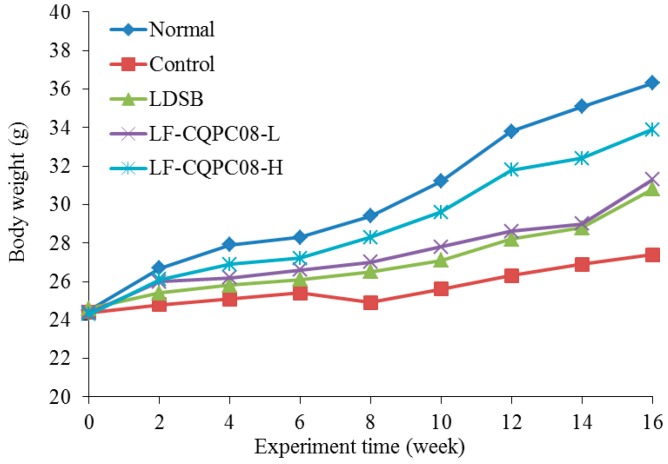
Body weight of mice during the experiment. LDSB: mice treated with 4NQO and *Lactobacillus delbruechii* subsp. *bulgaricus* (1.0 × 10^9^ CFU/kg); LF-CQPC08-L: mice treated with 4NQO and *Lactobacillus fermentum* CQPC08 (1.0 × 10^8^ CFU/kg); LF-CQPC08-H: mice treated with 4NQO and *Lactobacillus fermentum* CQPC08 (1.0 × 10^9^ CFU/kg).

*L. fermentum* are microorganisms approved by the US Food and Drug Administration (FDA) and China Food and Drug Administration (CFDA) for use in food, and they are safe to use. In previous experiments, all mice fed with LF-CQPC08 survived, and the IgG level was higher than that in normal mice without treatment with LF-CQPC08. In this study, all mice also survived during the experiment, and the weight of the tongue cancer mice was lower than that of the normal group (Figure 1); LF-CQPC08 could inhibit the body weight reduction in tongue cancer mice. Table 2 shows the splenic index and thymic index of the induced tongue cancer mouse model (Control group) to be significantly lower than that of the remaining groups of mice (*p* < 0.05). LF-CQPC08 significantly (*p* < 0.05) upregulated the splenic index and thymus index of the mice with induced tongue cancer, with the effects being better than the mice treated with same concentration of 4NQO and *Lactobacillus delbruechii* subsp. *bulgaricus* (LDSB). In addition, this up-regulatory effect of the splenic index and thymus index of the mice with induced tongue cancer was dose-dependent.

### 3.3. Effect of Lactobacillus Fermentum CQPC08 on Phagocytosis of Macrophages

Table 3 shows that the phagocytosis percentage and phagocytic index of the normal control mice (Normal group) were significantly higher than those of the remaining groups of mice (*p* < 0.05). LDSB and LF-CQPC08 significantly (*p* < 0.05) upregulated the phagocytosis percentage and phagocytic index of the mice with induced tongue cancer, and the effects of high-dose LF-CQPC08 (LF-CQPC08-H) were better than those of low-dose LF-CQPC08 (LF-CQPC08-L) and LDSB, and were similar to the results in the mice in the Normal group.

### 3.4. Mouse Serum Granulocyte-Colony Stimulating Factor (G-CSF), Granulocyte-Macrophage-CSF (GM-CSF), Immunoglobulin (Ig)G, and IgM Levels

As shown in Table 4, the serum G-CSF, GM-CSF, IgG, and IgM levels of the mice in the Normal group were the highest, while the results in the mice with induced tongue cancer in the control group were the opposite, showing the lowest serum levels. LF-CQPC08 significantly reduced the serum G-CSF, GM-CSF, IgG, and IgM levels, with the effects of the LF-CQPC08-H and LF-CQPC08-L group significantly better than those in the LDSB group (*p* < 0.05), and the effects of LF-CQPC08-H were better than those of LF-CQPC08-L.

### 3.5. Mouse Serum Interleukin (IL)-4, IL-12, Tumor Necrosis Factor-Alpha (TNF-A), and Interferon-Gamma (IFN-Γ) Levels

As shown in Table 5, the serum IL-4, IL-12, TNF-α, and IFN-γ levels of the mice in the normal group were the highest, while the results in the mice with induced tongue cancer in the control group were the opposite. LF-CQPC08 significantly upregulated serum IL-4, IL-12, TNF-α, and IFN-γ levels, with the effects of the LF-CQPC08-H group being better than those of the LF-CQPC08-L group. For the same concentration, the LF-CQPC08 had significantly better effects than the LDSB (*p* < 0.05).

### 3.6. Superoxide Dismutase (SOD) and Glutathione Peroxidase (GSH-Px) Activities and Malondialdehyde (MDA) Level in the Mouse Tongue Tissues

As shown in Table 6, the SOD and GSH-Px activities of the mice with induced tongue cancer in the Control group were the lowest, and the MDA level of the Control group was the highest among all groups of mice. After LDSB and LF-CQPC08 treatments, the SOD and GSH-Px activity levels were significantly elevated (*p* < 0.05), and the MDA level was significantly reduced (*p* < 0.05) in mice with induced tongue cancer. At the same concentration, the elevation of SOD and GSH-Px activities and the reduction of MDA level in the LF-CQPC08 group were more effective than that in the LDSB group.

### 3.7. mRNA Expression of p53, p63, p73, and Phosphatase and Tensin Homolog (PTEN) in the Mouse Tongue Tissues

As shown in Table 7, the mRNA expression of p53, p63, p73, and PTEN in the mouse tongue tissues of the control group was extremely weak, while the mRNA expression of p53, p63, p73, and PTEN in the mouse tongue tissues of the normal group were the strongest. After LDSB and LF-CQPC08 treatments, the expression of p53, p63, p73, and PTEN in the mouse tongue tissues were significantly downregulated. At the same concentration, the upregulation of the p53, p63, p73, and PTEN expression of the LF-CQPC08 group were stronger than in the LDSB group.

### 3.8. mRNA Expression of Nuclear Factor-Erythroid 2 Related Factor 2 (Nrf2), Heme Oxygenase-1 (HO-1), and Glutathione S-Transferase-Π (GST-Π) in the Mouse Tongue Tissues

As shown in Table 8, the mRNA expression of Nrf2, HO-1, and GST-π in the mouse tongue tissues of the control group was the strongest, while the mRNA expression of Nrf2, HO-1, and GST-π in the mouse tongue tissues of the Normal group were the weakest. LF-CQPC08 effectively upregulated the mRNA expression of Nrf2, HO-1, and GST-π in the tongue tissues of the mice with induced tongue cancer, with the similar effects found in the LF-CQPC08-H and LF-CQPC08-L groups. Relative to the mice with induced tongue cancer in the control group, the LF-CQPC08-H treatment greatly upregulated the mRNA expression of Nrf2, HO-1, and GST-π in the mouse town tissues, reaching nearly the levels of Nrf2, HO-1, and GST-π mRNA expression in the mouse tongue tissues of the normal group.

### 3.9. mRNA Expression of Bax, Bcl-2, and Bcl-Xl in the Mouse Tongue Tissues

As shown in Table 9, mRNA expression of Bax in the mouse tongue tissues of the Normal group was the strongest, and mRNA expression of Bcl-2 and Bcl-xL in the mouse tongue tissues of the Normal group was the weakest. In contrast, the mRNA expression of Bax in the mouse tongue tissues of the Control group was the weakest, and mRNA expression of Bcl-2 and Bcl-xL in the mouse tongue tissues of the control group was the strongest. Relative to the control group, LF-CQPC08 treatment upregulated the Bax mRNA expression and downregulated the Bcl-2 and Bcl-xL mRNA expression in the mouse tongue tissues; the result of the Bax mRNA expression in the mouse tongue tissues of the LF-CQPC08-H group was close to the control group and significantly higher than that of the LDSB group (*p* < 0.05); whereas the Bcl-2 and Bcl-xL mRNA expression in the mouse tongue tissues of the LF-CQPC08-H group was significantly lower than that of the LDSB group (*p* < 0.05).

## 4. Discussion

Recently, partial modulation of the human immune system against disease has been found to be a way of controlling some diseases. It involves enhancing immunity to prevent cancer cell invasion, suppress cancer, and use as adjuvant cancer therapy [26]. The splenic index, thymus index, phagocytosis percentage, and phagocytic index reflect the normal state of immune organs to a certain extent and directly reflect the strength of immune functions in the body. A previous study has shown that some active ingredients in food act as immunomodulators to avoid the effects of adverse factors on the immune organs, delay thymic degeneration, and maintain normal immunity [27]. The effect of lactic acid bacteria on non-specific immunity is mainly through enhancing the activity of mononuclear phagocytic cells (monocytes and macrophages) and polymorphonuclear leukocytes, stimulating the secretion of lysosomes and mononuclear factors, promoting the production of reactive oxygen and reactive nitrogen, and improving the phagocytosis of the mononuclear phagocytic system. Lactic acid bacteria stimulate specific immune responses mainly through the humoral immunity and the cell-mediated immunity [28]. The humoral immunity is achieved by producing antibodies in the body and increasing IgA, IgM, and IgG levels in mucosa and blood. Cell-mediated immunity is achieved by activating macrophages, B lymphocytes, and natural killer (NK) cells and by promoting the production of cytokines, such as IL and interferon (IFN) [29]. G-CSF and GM-CSF exert good therapeutic effects on neutropenia caused by cancer. They also have immunomodulatory and other effects on the number and proportion of T lymphocyte subsets in the process of hematopoietic stem/progenitor cells in the peripheral blood [30]. The experimental results of this study showed that LF-CQPC08 inhibited the effects of carcinogenic compounds on the splenic and thymic indexes in the mouse model. In addition, LF-CQPC08 improved the decline of serum G-CSF, GM-CSF, IgG, and IgM levels caused by oral cancer. The IgG, and IgM serum levels of normal mice in this study were similar to those in previous study [23]. Hence, LF-CQPC08 effectively inhibited the weakened immunity in the mice caused by tongue cancer and improved the immunity of the tumor-bearing mice, with a better effect than the LDSB commonly found on the market.

IL-12 plays an important role in enhancing the cell-mediated immunity and modulation of immune responses. It also stimulates the proliferation of activated T-cells, promotes the differentiation of Th0 cells into Th1 cells, induces cytotoxicity, and promotes the secretion of IFN-γ and GM-CSF of the NK cells, thereby exerting its inhibitory effects on cancer cells [31]. IL-4 is secreted by activated Th2 cells and promotes the proliferation and activation of B cells. IFN-γ is secreted by Th1 cells, inducing participation and enhancing cell-mediated immunity in the body. The ratio of Th1 and Th2 lymphocyte subsets is relatively constant, which is essential to maintaining the stability of immune functions [32]. IFN-γ also has antitumor effect and can promote autoimmunity [33]. TNF-α is mainly secreted by macrophages, and its main function is to regulate immune cells. The endogenous pyrogen TNF-α can cause fever and apoptosis and prevent tumorigenesis [34]. The inflammatory related cytokine serum levels of normal mice in this study were similar to those in a previous study [35]. In this study, LF-CQPC08 increased the serum levels of IL-4, IL-12, TNF-α, and IFN-γ in mice with induced tongue cancer, thereby regulating the immune system and inhibiting the tongue cancer in the mouse model.

The presence of large quantities of free radicals in the body promotes cell aging and death, damaging human organs and even DNA in human cells, causing cells to mutate, thereby inducing various diseases, including tumors. Cancer patients have a much lower antioxidant capacity than healthy individuals do, and the antioxidants prevent damage caused by cancer-induced and anticancer-treatment-induced elevation of free radical in normal cells [36]. Both SOD and GSH-Px are effective antioxidants that enhance the antioxidant capacity in the body, preventing and suppressing cancer [37]. MDA is one of the most important products of membrane lipid peroxidation. It causes cross-linking polymerization of macromolecules, such as proteins and nucleic acids, and it is cytotoxic, leading to further damage to the body. Thus, controlling the MDA levels in the body also prevents cancer [38]. In this study, LF-CQPC08 reduced oxidative stress damage in mice with tongue cancer and improved the SOD and GSH-Px activities and reduced the MDA level in the tongue tissues to protect the tongue tissues and lower the damage in the tongue tissue.

P53 plays an important role in the prevention of tumorigenesis. P73 and p63 are newly discovered members of the p53 family, and they have high homology in the sequences. Overexpression of p73 activates p53 and reactive promoter transcription causes cell growth and inhibits and induces apoptosis. One previous study showed that the expression of p73 is positively correlated with tumor metastasis and malignancy [39]. The structure and functions of p63 are more similar to those of p73 than to those of p53. p63 induces apoptosis in p53-deficient tumor cells. Clinical research has also shown that the positive expression rates of p63 and p53 in oral squamous cell carcinomas are significantly higher than in the normal oral mucosa [40]. Under normal conditions, p73, p63, and p53 are rarely expressed. In the development of oral cancers, the overexpression of p73, p63, and p53 proteins plays an important role in the pathogenesis of oral squamous cell carcinoma, and they all serve as tumor suppressor proteins. Among them, the interaction between p73 and p63 plays an important role in the development of oral squamous cell carcinomas [41]. PTEN is a tumor suppressor gene with phosphatase activity, and abnormal expression of PTEN was observed in many malignant tumors. Clinical research has shown that PTEN protein expression in oral squamous cell carcinomas is positively correlated with the degree of tissue differentiation [42]. In this study, the expression of p73, p63, p53, and PTEN in the normal mice was very weak. With the development of tongue cancer, the abundantly expressed p73, p63, p53, and PTEN exerted their anti-cancer roles. With the action of LF-CQPC08, the malignancy of tongue cancer was reduced, and the expression of p73, p63, p53, and PTEN was also decreased.

Imbalances in reactive oxygen species (ROS) and antioxidant systems lead to oxidative stress, which has been recognized as one of the carcinogenic factors. Nrf2 couples with Keap1 and binds to cytoplasmic actin, which anchors it in the cytoplasm. When a cell is subjected to oxidative stress, Nrf2 is uncoupled from Keap1 and translocated into nuclei. Nrf2 is recognized after binding to Maf protein to form a heterodimer, which combines with antioxidant response elements (ARE) to initiate the gene transcription of phase 2 detoxification enzymes, i.e., GST, NAD(P)H-quinone oxidoreductase 1 (NQO1), SOD, HO-1, and glutamate-cysteine ligase (GCL) and antioxidative stress proteins, thereby improving cells’ ability to resist oxidative stress [43]. HO-1, which is one of the most widely occurring antioxidant defense enzymes, has antioxidant and anti-inflammatory effects. GST-π is also an important phase 2 detoxification enzyme that inhibits cancer through its antioxidant effects [44]. In the case of non-overexpression, Nrf2, HO-1, and GST-π exert their antioxidant effects and inhibit oral cancer [45]. In this study, LF-CQPC08 played a role in regulating the overexpression of Nrf2, HO-1, and GST-π in the tissues of the tongue and inhibited the development of oral cancer, with better therapeutic effect than LDSB.

In mammalian cells, the regulation of mitochondrial extracorporeal membrane permeability occurs mostly on the extracorporeal membrane of mitochondria, or transfers to the extracorporeal membrane of mitochondria after being stimulated by signals. These molecules are divided into two groups, according to their functions: (1) anti-apoptotic proteins, such as Bcl-2, Bcl-xL, and Bcl-w; and (2) pro-apoptotic proteins, such as Bax, Bak, and Noxa [46]. In oral cancer, Bcl-2 and Bcl-xL expression plays a role in promoting tumor growth, and Bax expression plays a role in suppressing the cancer [47]. In this study, both LF-CQPC08 and LDSB effectively inhibited the expression of Bcl-2 and Bcl-xL and increased the expression of Bax, thereby exerting their inhibitory effects on the oral cancer in the mice. The inhibitory effect of the LF-CQPC08 was stronger than that of the LDSB.

## 5. Conclusions

Lactic acid bacteria may prevent intestinal cancer via several mechanisms: binding to and degrading potential carcinogens, producing anti-cancer substances, inhibiting tumor growth, and improving immunity. Using animal experiments to test the activity of functional foods can lay a foundation for human clinical trials. In this study, animal models were used to simulate the status of human tongue cancer. The newly isolated and identified lactic acid bacteria, LF-CQPC08, were tested in vivo to observe their biological activities against tongue cancer. The qPCR results showed that there were great differences between the mRNA expression of tongue cancer mice and the normal state. Visual observation also showed that the tongue of mice had lumps, suggesting that the tongue cancer model was successful. The experimental results showed that LF-CQPC08 could prevent the 4NQO-induced experimental tongue cancer effectively and improved the immunity and antioxidation ability of the mice with tongue cancer, thereby alleviating the effects of experimental tongue cancer on mice. The effects of LF-CQPC08 on tongue cancer in mice might be due to improvements in immunity of the animal body or its metabolites. In addition, the inhibitory effects of LF-CQPC08 on the oxidative stress damage in the mouse model might help repair the damage caused by tongue cancer, thereby inhibiting tongue cancer. LF-CQPC08, a newly discovered bacterial strain, exhibits a better effect on oral cancer than the bacterial strain LDSB, a strain commonly used in the food industry. Thus, LF-CQPC08 may be a high-quality bacterial strain with biological activity and possess application values in food and pharmaceutical industries. To better apply LF-CQPC08, further research should focus on establishing the physiological mechanisms of LF-CQPC08.

## Figures and Tables

**Table 1:** Sequences of primers used in this study.

Gene Name	Sequence
p53	Forward: 5′-TAACAGTTCCTGCATGGGCGGC-3′
	Reverse: 5′-AGGACAGGCACAAACACGCACC-3′
p63	Forward: 5′-GTCAGCCACCTGGACGTATT-3′
	Reverse: 5′-ACCTGTGGTGGCTCATAAGG-3′
p73	Forward: 5′-GAGCCTTTGGTTGACTCCT-3′
	Reverse: 5′-CCACCGTGTACCTTGTTCAT-3′
PTEN	Forward: 5′-TGGAAAGGGACGAACTGGTG-3′
	Reverse: 5′-CATAGCGCCTCTGACTGGGA-3′
Nrf2	Forward: 5′-CAGTGCTCCTATGCGTGAA-3′
	Reverse: 5′-GCGGCTTGAATGTTTGTC-3′
HO-1	Forward: 5′-ACAGATGGCGTCACTTCG-3′
	Reverse: 5′-TGAGGACCCACTGGAGGA-3′
GST-π	Forward: 5′-CTCTGTCTACGCAGCACTGAATC-3′
	Reverse: 5′-CAAGCCTTGCATCCAGGTATC-3′
Bax	Forward: 5′-TTGCTACAGGGTTTCATC-3′
	Reverse: 5′-TCCAGTTCATCTCCAATTC-3′
Bcl-2	Forward: 5′-GAGATCGTGATGAAGTACATAC-3′
	Reverse: 5′-TCAGGCTGGAAGGAGAAG-3′
Bcl-xL	Forward: 5′-CTTTCGGGATGGAGTAAAC-3′
	Reverse: 5′-AGGTGGTCATTCAGATAGG-3′
GAPDH	Forward: 5′-AGGTCGGTGTGAACGGATTTG-3′
	Reverse: 5′-GGGGTCGTTGATGGCAACA-3′

PTEN: phosphatase and tensin homolog; Nrf2: nuclear factor-erythroid 2 related factor 2; HO-1: heme oxygenase-1; GST-π: glutathione-S-transferases-π; Bax: Bcl-2-associated X protein; Bcl-2: B-cell lymphoma 2; Bcl-xL: B-cell lymphoma-extra large; GAPDH: glyceraldehyde-3-phosphate dehydrogenase.

**Table 2:** Effect of *Lactobacillus fermentum* CQPC08 on immune organ index in mice with tongue cancer.

Group	Spleen Index	Thymus Index
Normal	5.33 ± 0.31 ^a^	2.52 ± 0.15 ^a^
Control	2.69 ± 0.28 ^d^	1.36 ± 0.10 ^c^
LDSB	3.58 ± 0.21 ^c^	1.79 ± 0.09 ^b^
LF-CQPC08-L	3.67 ± 0.33 ^c^	1.85 ± 0.13 ^b^
LF-CQPC08-H	4.69 ± 0.24 ^b^	1.93 ± 0.11 ^b^

Values presented are the mean ± standard deviation (N = 10/group). ^a–^^d^ Mean values with different letters in the same row are significantly different (*p* < 0.05) according to Duncan’s multiple-range test. LDSB: mice treated with 4NQO and *Lactobacillus delbruechii* subsp. *bulgaricus* (1.0 × 10^9^ CFU/kg); LF-CQPC08-L: mice treated with 4NQO and *Lactobacillus fermentum* CQPC08 (1.0 × 10^8^ CFU/kg); LF-CQPC08-H: mice treated with 4NQO and *Lactobacillus fermentum* CQPC08 (1.0 × 10^9^ CFU/kg).

**Table 3:** Effect of *Lactobacillus fermentum* CQPC08 on phagocytosis of macrophages in mice with tongue cancer.

Group	Phagocytic Percentage (%)	Phagocytic Index
Normal	37.62 ± 2.55 ^a^	1.71 ± 0.18 ^a^
Control	16.31 ± 2.03 ^d^	0.56 ± 0.07 ^d^
LDSB	22.17 ± 2.19 ^c^	0.94 ± 0.08 ^c^
LF-CQPC08-L	23.02 ± 2.67 ^c^	0.98 ± 0.11 ^c^
LF-CQPC08-H	30.12 ± 1.74 ^b^	1.39 ± 0.12 ^b^

Values presented are the mean ± standard deviation (N = 10/group). ^a–^^d^ Mean values with different letters in the same row are significantly different (*p* < 0.05) according to Duncan’s multiple-range test. LDSB: mice treated with 4NQO and *Lactobacillus delbruechii* subsp. *bulgaricus* (1.0 × 10^9^ CFU/kg); LF-CQPC08-L: mice treated with 4NQO and *Lactobacillus fermentum* CQPC08 (1.0 × 10^8^ CFU/kg); LF-CQPC08-H: mice treated with 4NQO and *Lactobacillus fermentum* CQPC08 (1.0 × 10^9^ CFU/kg).

**Table 4:** Serum levels of G-CSF, GM-CSF, IgG and IgM in mice.

Group	G-CSF (pg/mL)	GM-CSF (pg/mL)	IgG (μg/mL)	IgM (μg/mL)
Normal	557.28 ± 21.05 ^a^	25.87 ± 1.03 ^a^	536.79 ± 15.62 ^a^	5.87 ± 0.25 ^a^
Control	218.67 ± 14.32 ^d^	14.32 ± 0.49 ^d^	234.15 ± 12.17 ^d^	2.61 ± 0.13 ^d^
LDSB	341.28 ± 18.97 ^c^	18.43 ± 0.51 ^c^	368.36 ± 13.25 ^c^	3.77 ± 0.21 ^c^
LF-CQPC08-L	352.17 ± 22.08 ^c^	18.89 ± 0.44 ^c^	377.21 ± 16.23 ^c^	3.89 ± 0.23 ^c^
LF-CQPC08-H	435.64 ± 24.52 ^b^	22.08 ± 0.36 ^b^	450.22 ± 15.20 ^b^	4.97 ± 0.18 ^b^

Values presented are the mean ± standard deviation (N = 10/group). ^a–^^d^ Mean values with different letters in the same row are significantly different (*p* < 0.05) according to Duncan’s multiple-range test. LDSB: mice treated with 4NQO and *Lactobacillus delbruechii* subsp. *bulgaricus* (1.0 × 10^9^ CFU/kg); LF-CQPC08-L: mice treated with 4NQO and *Lactobacillus fermentum* CQPC08 (1.0 × 10^8^ CFU/kg); LF-CQPC08-H: mice treated with 4NQO and *Lactobacillus fermentum* CQPC08 (1.0 × 10^9^ CFU/kg). G-CSF: granulocyte-colony stimulating factor; GM-CSF: granulocyte-macrophage colony-stimulating factor; IgG: immunoglobulin G; IgM: immunoglobulin M.

**Table 5:** Serum cytokines levels of IL-4, IL-12, TNF-α and IFN-γ in mice.

Group	IL-4 (pg/mL)	IL-12 (pg/mL)	TNF-α (pg/mL)	IFN-γ (pg/mL)
Normal	67.32 ± 2.56 ^e^	733.25 ± 22.59 ^a^	163.58 ± 10.11 ^a^	155.12 ± 11.05 ^a^
Control	30.58 ± 2.29 ^a^	162.37 ± 12.15 ^d^	31.05 ± 2.32 ^d^	29.36 ± 4.32 ^d^
LDSB	42.12 ± 3.15 ^d^	255.36 ± 25.30 ^c^	78.20 ± 9.52 ^c^	55.21 ± 5.36 ^c^
LF-CQPC08-L	43.25 ± 3.08 ^b^	259.17 ± 31.02 ^c^	81.98 ± 8.35 ^c^	57.89 ± 8.36 ^c^
LF-CQPC08-H	55.29 ± 2.91 ^c^	452.84 ± 26.34 ^b^	118.05 ± 5.13 ^b^	105.28 ± 4.11 ^b^

Values presented are the mean ± standard deviation (N = 10/group). ^a–^^d^ Mean values with different letters in the same row are significantly different (*p* < 0.05) according to Duncan’s multiple-range test. LDSB: mice treated with 4NQO and *Lactobacillus delbruechii* subsp. *bulgaricus* (1.0 × 10^9^ CFU/kg); LF-CQPC08-L: mice treated with 4NQO and *Lactobacillus fermentum* CQPC08 (1.0 × 10^8^ CFU/kg); LF-CQPC08-H: mice treated with 4NQO and *Lactobacillus fermentum* CQPC08 (1.0 × 10^9^ CFU/kg). IL-4: interleukin-4; IL-12: interleukin-12; TNF-α: tumor necrosis factor-α; IFN-γ: interferon-γ.

**Table 6:** Tongue tissue levels of SOD, GSH-Px and MDA in mice.

Group	SOD (U/mgprot)	GSH-Px (U/mgprot)	MDA (nmol/mgprot)
Normal	73.52 ± 4.31 ^a^	123.05 ± 7.82 ^a^	0.88 ± 0.07 ^d^
Control	25.77 ± 2.19 ^d^	46.78 ± 3.54 ^d^	3.72 ± 0.23 ^a^
LDSB	48.37 ± 3.66 ^c^	74.23 ± 5.22 ^c^	2.25 ± 0.28 ^b^
LF-CQPC08-L	49.69 ± 4.02 ^c^	76.15 ± 5.31 ^c^	2.11 ± 0.25 ^b^
LF-CQPC08-H	60.87 ± 4.33 ^b^	97.82 ± 6.28 ^b^	1.42 ± 0.18 ^c^

Values presented are the mean ± standard deviation (N = 10/group). ^a–^^d^ Mean values with different letters in the same row are significantly different (*p* < 0.05) according to Duncan’s multiple-range test. LDSB: mice treated with 4NQO and *Lactobacillus delbruechii* subsp. *bulgaricus* (1.0 × 10^9^ CFU/kg); LF-CQPC08-L: mice treated with 4NQO and *Lactobacillus fermentum* CQPC08 (1.0 × 10^8^ CFU/kg); LF-CQPC08-H: mice treated with 4NQO and *Lactobacillus fermentum* CQPC08 (1.0 × 10^9^ CFU/kg). SOD: superoxide dismutase; GSH-Px: glutathione peroxidase; MDA: malondialdehyde.

**Table 7:** p53, p63, p73 and PTEN mRNA expression in tongue tissue of mice (relative to multiple of control group).

Group	p53	p63	p73	PTEN
Normal	0.11 ± 0.02 ^d^	0.09 ± 0.03 ^d^	0.18 ± 0.02 ^d^	0.32 ± 0.03 ^d^
Control	1.00 ± 0.06 ^a^	1.00 ± 0.04 ^a^	1.00 ± 0.07 ^a^	1.00 ± 0.04 ^a^
LDSB	0.65 ± 0.04 ^b^	0.78 ± 0.04 ^b^	0.62 ± 0.05 ^b^	0.75 ± 0.03 ^b^
LF-CQPC08-L	0.63 ± 0.03 ^b^	0.75 ± 0.04 ^b^	0.59 ± 0.06 ^b^	0.67 ± 0.04 ^b^
LF-CQPC08-H	0.29 ± 0.03 ^c^	0.58 ± 0.03 ^c^	0.31 ± 0.05 ^c^	0.48 ± 0.04 ^c^

Values presented are the mean ± standard deviation (N = 10/group). ^a–^^d^ Mean values with different letters in the same row are significantly different (*p* < 0.05) according to Duncan’s multiple-range test. LDSB: mice treated with 4NQO and *Lactobacillus delbruechii* subsp. *bulgaricus* (1.0 × 10^9^ CFU/kg); LF-CQPC08-L: mice treated with 4NQO and *Lactobacillus fermentum* CQPC08 (1.0 × 10^8^ CFU/kg); LF-CQPC08-H: mice treated with 4NQO and *Lactobacillus fermentum* CQPC08 (1.0 × 10^9^ CFU/kg).

**Table 8:** Nrf2, HO-1, and GST-π mRNA expression in tongue tissue of mice (relative to multiple of control group).

Group	Nrf2	HO-1	GST-π
Normal	4.02 ± 0.25 ^a^	4.52 ± 0.15 ^a^	5.87 ± 0.21 ^a^
Control	1.00 ± 0.12 ^d^	1.00 ± 0.05 ^d^	1.00 ± 0.16 ^d^
LDSB	2.38 ± 0.15 ^c^	2.67 ± 0.18 ^c^	3.66 ± 0.25 ^c^
LF-CQPC08-L	2.49 ± 0.26 ^c^	2.81 ± 0.16 ^c^	3.81 ± 0.23 ^c^
LF-CQPC08-H	3.11 ± 0.15 ^b^	3.66 ± 0.22 ^b^	4.98 ± 0.29 ^b^

Values presented are the mean ± standard deviation (N = 10/group). ^a–^^d^ Mean values with different letters in the same row are significantly different (*p* < 0.05) according to Duncan’s multiple-range test. LDSB: mice treated with 4NQO and *Lactobacillus delbruechii* subsp. *bulgaricus* (1.0 × 10^9^ CFU/kg); LF-CQPC08-L: mice treated with 4NQO and *Lactobacillus fermentum* CQPC08 (1.0 × 10^8^ CFU/kg); LF-CQPC08-H: mice treated with 4NQO and *Lactobacillus fermentum* CQPC08 (1.0 × 10^9^ CFU/kg).

**Table 9:** Nrf2, HO-1, and GST-π mRNA expression in tongue tissue of mice (relative to multiple of control group).

Group	Bax	Bcl-2	Bcl-xL
Normal	8.21 ± 0.36 ^a^	0.35 ± 0.03 ^d^	0.26 ± 0.05 ^d^
Control	1.00 ± 0.25 ^d^	1.00 ± 0.06 ^a^	1.00 ± 0.07 ^a^
LDSB	4.87 ± 0.32 ^c^	0.77 ± 0.05 ^b^	0.69 ± 0.02 ^b^
LF-CQPC08-L	5.03 ± 0.24 ^c^	0.72 ± 0.06 ^b^	0.68 ± 0.05 ^b^
LF-CQPC08-H	6.79 ± 0.39 ^b^	0.54 ± 0.04 ^c^	0.45 ± 0.04 ^c^

Values presented are the mean ± standard deviation (N = 10/group). ^a–^^d^ Mean values with different letters in the same row are significantly different (*p* < 0.05) according to Duncan’s multiple-range test. LDSB: mice treated with 4NQO and *Lactobacillus delbruechii* subsp. *bulgaricus* (1.0 × 10^9^ CFU/kg); LF-CQPC08-L: mice treated with 4NQO and *Lactobacillus fermentum* CQPC08 (1.0 × 10^8^ CFU/kg); LF-CQPC08-H: mice treated with 4NQO and *Lactobacillus fermentum* CQPC08 (1.0 × 10^9^ CFU/kg).
